# Paper-Based Biosensor Using Dual-Recognition Molecules for Detection of *Staphylococcus aureus* in Milk

**DOI:** 10.3390/bios16080417

**Published:** 2026-08-03

**Authors:** Weichen Hou, Xiangyang Li, Jie Li, Kai Dong, Wen Zhao, Zhaozhong Zeng, Jian He, Longjiao Zhu, Wentao Xu

**Affiliations:** 1 Food Laboratory of Zhongyuan, Key Laboratory of Precision Nutrition and Food Quality, Department of Nutrition and Health, China Agricultural University, Beijing 100193, Chinaxuwentao@cau.edu.cn (W.X.); 2 Food Science and Engineering College, Beijing University of Agriculture, Beijing 102206, China; 3 National Center of Technology Innovation for Dairy, Hohhot 010105, China

**Keywords:** *Staphylococcus aureus*, aptamer, antibodies, paper-based biosensor, rapid detection

## Abstract

*Staphylococcus aureus* (*S. aureus*) is a common foodborne pathogen that can cause the severe contamination of dairy products. Therefore, there is an urgent need for rapid detection methods. In this study, a paper-based biosensor integrating a dual-recognition strategy using aptamers and antibodies was developed for the sensitive, rapid, and on-site detection of *S. aureus* in complex milk matrices. The biosensor combines a milk matrix-adapted aptamer with polyclonal antibodies (pAbs) and utilizes colloidal gold nanoparticles (AuNPs) as visual signal reporters. Using a SELEX process tailored to the milk matrix, the high-affinity aptamer SA2-1 was selected to specifically bind *S. aureus*, while pAbs enabled multi-epitope capture on the paper substrate. The aptamer–AuNP conjugates generated visual signals, achieving a detection limit of 10^2^ CFU/mL within 15 min without an instrument. This dual-recognition strategy synergistically enhances both sensitivity and specificity, offering a cost-effective solution for dairy safety monitoring.

## 1. Introduction

*Staphylococcus aureus* (*S. aureus*) is a major foodborne pathogen responsible for the contamination of dairy products [1]. It is also a primary infectious agent in bovine mastitis and various pyogenic infections, posing significant contamination risks in dairy products [2,3]. As an opportunistic pathogen, *S. aureus* predominantly colonizes the nostrils, skin, and hair follicles of warm-blooded mammals [4,5]. This risk is further exacerbated in developing countries, where inadequate cold-chain infrastructure facilitates the growth and spread of the pathogen. Following the ingestion of food contaminated with *S. aureus*, symptoms of intoxication typically emerge within 0.5–8 h [6]. Clinical manifestations include nausea, vomiting, and watery diarrhea, often accompanied by abdominal cramps and fever. In severe cases, life-threatening dehydration and electrolyte imbalances may occur [7]. Therefore, the development of rapid and sensitive methodologies suitable for on-site detection is imperative for safeguarding dairy product safety and public health.

However, conventional detection methods predominantly rely on labor-intensive and time-consuming plate culture techniques [8,9]. Polymerase chain reaction (PCR), while effective, faces limitations in resource-constrained environments due to its dependence on expensive instrumentation and specialized personnel. Immunoassays like the enzyme-linked immunosorbent assay (ELISA) offer faster detection, but they suffer from challenges including batch-to-batch antibody variability and limited target accessibility [10]. In contrast, paper-based lateral flow assay (LFA) methods have emerged as a promising alternative for on-site food safety monitoring, as they enable visual result interpretation within minutes and require no specialized personnel to operate [11,12,13].

In recent years, aptamers have emerged as promising novel recognition molecules [14]. These short single-stranded DNA or RNA oligonucleotides demonstrate high specificity, chemical stability, and ease of synthesis, making them attractive alternatives to traditional antibodies [15]. Aptamers are typically selected through the Systematic Evolution of Ligands by Exponential Enrichment (SELEX) process, which enables the generation of high-affinity sequences [16]. Notably, aptamer properties vary with the screening conditions and background environment [17]. This allows researchers to optimize their performance by introducing target matrices directly into the SELEX process, a strategy that enhances adaptability in complex sample environments [18]. Among various SELEX strategies, cell-SELEX preserves the native conformations of surface epitopes and is especially suitable for whole-cell targeting applications [19]. Although antibodies are widely used due to their proven clinical utility and established production platforms, aptamers offer notable advantages, including higher chemical stability, batch-to-batch consistency, and the ability to be easily synthesized and modified in vitro. Nevertheless, both recognition systems exhibit inherent limitations. Antibody-based detection methods suffer from unreliable performance, including insufficient lot-to-lot consistency and thermal instability—issues that have even led to false findings and wasted efforts in related studies [20]. On the other hand, traditional aptamers may form secondary or tertiary structures due to their relatively long sequences, which can compromise the detection performance of aptasensors in real-sample analysis [21]. These dual challenges make single-recognition molecules, whether antibodies or aptamers, highly susceptible to false positives or false negatives. To resolve these complementary shortcomings, integrated systems combining antibodies and aptamers have shown enhanced diagnostic capabilities. Recent antibody–aptamer dual-recognition systems have been widely validated across diverse analytical targets. One study utilized antibody-functionalized magnetic beads to specifically capture *Escherichia coli* O157:H7, pairing them with aptamer–gold nanoparticle probes for sensitive fluorescence signal generation [22]. This sandwich configuration has also been successfully adapted into chemiluminescent biosensors for the highly precise detection of aflatoxin B1 [23]. Additionally, hybrid lateral flow strips and enzyme-linked sandwich assays have been engineered for the rapid screening of SARS-CoV-2 in clinical samples [24]. This dual-recognition mode effectively mitigates the epitope overlap problem inherent in single-recognition molecules while ensuring robust recognition specificity. However, the integration of antibody–aptamer sandwich assays with paper-based biosensors for the detection of large pathogenic bacteria has yet to be reported, and achieving reliable performance within complex food matrices remains a formidable challenge.

To overcome the limitations of single-recognition biosensors, this study presents a paper-based lateral flow biosensor integrating tailored aptamers with polyclonal antibodies for the rapid detection of *S. aureus* in milk. Through the rational truncation of the parent aptamer sequence, we identified a conserved core region exhibiting enhanced binding affinity to the target pathogen. We then carried out rational library design to screen for aptamers with improved affinity and stability under milk matrix conditions, thereby reducing non-specific adsorption. The biosensor incorporates nitrocellulose-immobilized polyclonal antibodies as capture elements, while aptamer-conjugated colloidal gold nanoparticles (AuNPs) function as visual signal probes. This dual-recognition system provides distinct synergistic benefits. Polyclonal antibodies bind to multiple surface epitopes to offset potential epitope mutations and lower the risk of false-negative results, while aptamers enhance specificity and anti-interference capabilities in complex dairy matrices. Aptamer–AuNP conjugates produce a visible red band on the test line via nanoparticle aggregation, allowing result interpretation without instrumentation. This paper-based format enables the portable, rapid, and cost-effective screening of dairy products, making it highly suitable for on-site food safety monitoring in resource-limited settings.

## 2. Materials and Methods

### 2.1. Materials

Bovine serum albumin (BSA) was purchased from Sinopharm Chemical Reagent Co., Ltd. (Shanghai, China). Universal SYBR qPCR Mix was obtained from Beijing Ruibo Xingke Technology Co., Ltd. (Beijing, China). dNTPs and PerfectStart^®^ Top Green qPCR SuperMix were sourced from TransGen Biotech Co., Ltd. (Beijing, China). Aptamer sequences, the DNA oligonucleotide library, and SELEX forward/reverse primers were purchased from Shanghai Sangon Biotechnology Co., Ltd. (Shanghai, China). The tailored aptamer sequences designed in this study and the selected sequences after screening are listed in Tables S1 and S2.

Rabbit-derived polyclonal IgG antibodies and bacterial strains, including *S. aureus* (ATCC 29213), *Bacillus cereus* (*B. cereus*), *Enterobacter sakazakii* (*E. sakazakii*), *Salmonella enteritidis* (*S. enteritidis*), *Listeria monocytogenes* (*L. monocytogenes*), and *Escherichia coli* O157:H7 (*E. coli* O157:H7), were provided by the Key Laboratory of Agri-Food Safety Evaluation (Ministry of Agriculture and Rural Affairs), China Agricultural University (Beijing, China). Additional Staphylococcus strains, including *S. haemolyticus*, *S. epidermidis*, and *S. hominis*, were supplied by the Second Affiliated Hospital of Anhui Medical University (Hefei, China).

### 2.2. Aptamer Tailoring

The synthesized aptamer was diluted to 100 μM and serially diluted to establish a concentration gradient. Amplification reactions were performed on a real-time quantitative PCR (qPCR) system to generate a standard curve [25]. Then, 990 μL of *S. aureus* culture at a concentration of 10^7^ CFU/mL was centrifuged, resuspended in binding buffer (50 mM Tris, 15 mM NaCl, 5 mM MgCl_2_·6H_2_O, pH 7.5), and subsequently mixed with 10 μL 100 nM of heat-denatured aptamer solution. The mixture was incubated at 25 °C for 1 h, followed by four sequential washes with binding buffer. *S. aureus* was then resuspended in 100 μL of binding buffer, subjected to thermal denaturation, and centrifuged. The resultant supernatant was collected as a qPCR template. The 20 μL reaction mixture contained 1 μL template DNA, 0.4 μL each of the 10 μM forward and reverse primers, 10 μL qPCR SuperMix, and 8.2 μL nuclease-free water. The thermal cycling conditions included an initial denaturation step at 95 °C for 30 s, followed by 40 cycles of denaturation at 95 °C for 5 s and combined annealing at 60 °C for 30 s.

### 2.3. Milk Matrix-Adapted Aptamer SELEX

Based on the core sequence obtained from the previous tailoring step, a rationally designed aptamer library was constructed and subsequently subjected to SELEX against intact *S. aureus* cells [26]. To ensure specificity, *B. cereus*, *E. sakazakii*, *S. enteritidis*, *L. monocytogenes*, and *E. coli* O157:H7 were employed as negative selection targets. Primer Tm values were calculated using the NEB online tool (https://ligasecalc.neb.com/#!/ligation, accessed on 24 June 2026), and the optimized annealing temperature range was determined to be 54.5–65.5 °C. The aptamer library was incubated with *S. aureus* following the same procedure used to obtain the PCR template. The PCR reaction system consisted of 1.5 μL 10 μM forward primer, 1.5 μL 10 μM reverse primer, 5 μL 10× PCR buffer, 1 μL 10 mM dNTPs, 0.5 μL rTaq polymerase, 10 μL 100 nM template, and 30.5 μL RNase-free water. Negative SELEX steps were incorporated into rounds 4, 7, and 9. These steps involved the incubation of denatured libraries with negative strains (e.g., *B. cereus*, all at a concentration of 10^7^ CFU/mL), followed by centrifugation (6000× *g*, 4 °C, 5 min). The supernatant containing unbound aptamers was collected for subsequent positive SELEX. After PCR amplification, the products were treated with Lambda exonuclease at 37 °C for 5 to 60 min to generate single-stranded DNA. The reaction was then inactivated by heating at 75 °C for 10 min. Digested DNA was purified via ethanol precipitation and redissolved in ultrapure water for the next SELEX cycle. SELEX under milk matrix conditions was initiated from round 8 onward, and sequencing analysis was conducted after round 11.

### 2.4. Aptamer Evaluation

FAM-labeled aptamers were incubated with *S. aureus*. Flow cytometry was used to quantify the proportion of cells in positive samples with a fluorescence intensity exceeding that of the negative control group, and the dissociation constants (Kd) of the aptamers were calculated by non-linear fitting analysis according to the equation Y = Bmax × X/(Kd + X). In this equation, Y is the measured fluorescence intensity, and X is the aptamer concentration. The specificity assessment involved the parallel incubation of the selected aptamer with *B. cereus*, *E. sakazakii*, *L. monocytogenes*, *E. coli* O157:H7, *S. haemolyticus*, *S. epidermidis*, and *S. hominis*. Quantitative analysis was performed using the qPCR method detailed in Section 2.3, incorporating standard curve generation and cycle threshold determination.

### 2.5. Preparation of Aptamer–AuNP Probes

The experimental process for modifying thiolated aptamers onto AuNPs employed microwave-assisted heating [27]. Thiol-modified aptamers activated with 10 μM of TCEP were added to 400 μL of 4× concentrated colloidal gold solution. The mixture was heated in a microwave oven at a power input of 1150 W and output of 700 W (medium–high fire setting) for 3 min. After heating, PBS buffer (pH 7.4) was added, and the mixture was centrifuged. The resulting pellet was resuspended to a final volume of 400 μL. Subsequently, 100 μL of 10% BSA was added to the suspension, which was then incubated for 30 min. Following incubation, the mixture was centrifuged at 14,000× *g* rpm for 20 min, and the supernatant was discarded. The obtained pellet was finally resuspended in reconstitution buffer (20 mM Na_3_PO_4_, 0.25% Tween 20, 10% sucrose) for storage.

### 2.6. Assembly of Paper-Based Biosensors

Using the BioDot-XYZ3060 (BioDot, Inc., Irvine, CA, USA) dispensing system, different concentrations of pAb were precisely dispensed onto the T-line of the nitrocellulose (NC) membrane. The biotinylated aptamer complementary strands incubated for 1 h and 1 mg/mL streptavidin were dispensed onto the C-line of the NC membrane. The fixed distance between the T-line and C-line was 5 mm, and the membrane was dried at 37 °C for 12 h for later use. The sample pad, NC membrane, and absorbent paper were sequentially pasted onto the PVC backing plate. A programmable strip cutter was used to uniformly cut the assembled test strips to a width of 3.5 mm. The assembled paper-based biosensors were sealed in self-sealing bags and stored under vacuum with desiccants.

### 2.7. Sensitivity and Specificity Detection

*S. aureus* at 10^7^ CFU/mL was centrifuged, washed with binding buffer, and diluted to different concentrations. Then, 10 μL of the bacterial suspension was mixed with 20 μL of the reconstitution buffer for aptamer–AuNP probes for 10 min, followed by the addition of 30 μL of running buffer for mixing. The mixture was then dropped onto the sample pad and allowed to run for 15 min. Using the same method, the specific strains mentioned in Section 2.4 at 10^5^ CFU/mL were tested. The grayscale values of all T-lines were captured using the ImageJ 1.47 software, and the relative grayscale of each band was calculated by subtracting the background grayscale to quantify the gray intensity in order to evaluate the sensitivity and specificity. Each experimental data point was derived from three parallel samples.

### 2.8. Milk Sample Detection

*S. aureus* at 10^7^ CFU/mL was inoculated into commercially available skimmed milk. The milk containing the bacterial solution was incubated for 5 h, serially diluted tenfold to prepare three samples, and analyzed using the plate method. Then, 10 μL of the milk containing the bacterial solution was mixed with 50 μL of running buffer, and detection was performed using the plate culture method and the test strips, respectively. Each experimental data point was derived from three parallel samples.

## 3. Results and Discussion

### 3.1. Tailoring of High-Affinity Aptamers

**Figure 1:**
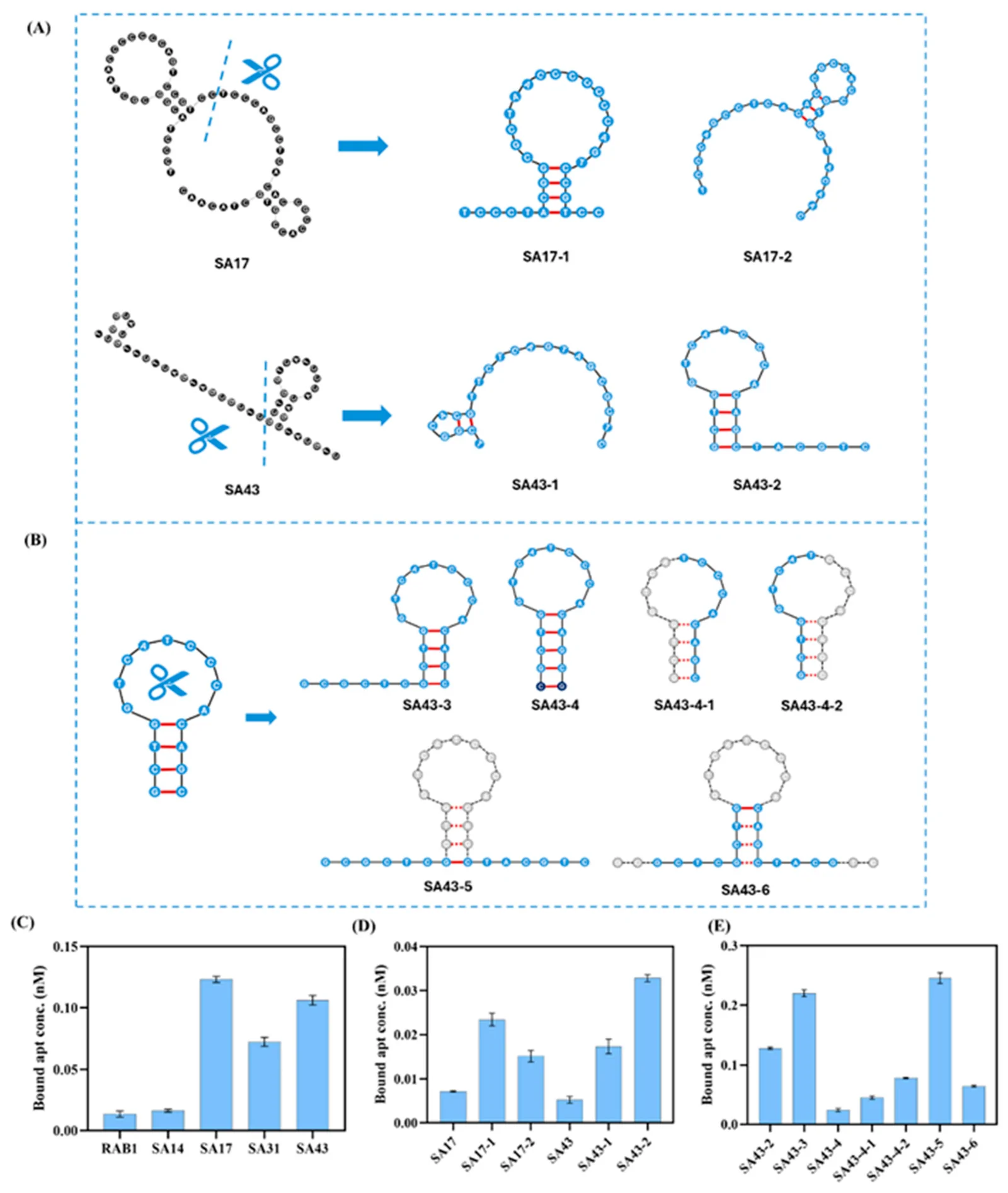
Tailoring of aptamer sequences to enhance binding affinity toward *S. aureus*. (**A**) Schematic representation of the primary tailoring of parent aptamers SA17 and SA43. (**B**) Schematic representation of the secondary tailoring of SA43-derived sequences based on stem-loop structures. (**C**) Comparative binding concentrations of five candidate aptamers to *S. aureus*. (**D**) Binding concentration analysis of primary tailoring aptamers evaluated via qPCR. (**E**) Binding concentration profiles of secondary tailoring SA43 variants. Error bars indicate standard deviations from three independent technical replicates. Absolute concentration variations across (**C**–**E**) are due to inter-batch differences.

Although a variety of *S. aureus*-specific aptamers have been identified, several studies have indicated that full-length aptamer sequences obtained through the SELEX often contain non-specific binding regions or base mismatches, resulting in redundant nucleotides [28]. These redundant segments do not contribute to target binding and may even interfere with affinity. Therefore, higher-affinity aptamers can be obtained by performing targeted tailoring on longer aptamers and retaining the most core bases of the aptamers. In this study, five commonly reported *S. aureus* aptamer sequences (Table 1) were selected, and their binding abilities to the target strain were compared (Figure 1C). Among them, SA17 and SA43 showed relatively strong binding performance and were chosen as the basis for subsequent tailoring optimization. The secondary structures of these two sequences were analyzed using the DNAMAN 9.0 software and served as the basis for truncation design.

In the initial tailoring experiment, SA17 and SA43 were split at their central positions, generating four truncated fragments designated as SA17-1, SA17-2, SA43-1, and SA43-2 (Figure 1A). Comparing the four tailored fragments with their pre-tailored aptamers, the 23 nt sequence near the 3′ end of SA43 displayed significantly enhanced binding affinity (Figure 1D). Subsequently, SA43-2 was used as the basis for further optimization. Meanwhile, secondary structure analysis of SA43 revealed the presence of a 17 nt stem–loop structure located between nucleotides 24 and 40. The region corresponding to SA43-2 included this stem–loop along with 6 nt at the 3′ end.

To investigate the role of the stem–loop, a second round of tailoring focused on the stem–loop and its extended flanking regions (Figure 1B). The 6 nt at the 5′ end of the stem–loop, symmetrical to SA43-2, were designated as SA43-3. In parallel, the stem–loop and its flanking regions were separated and designated as SA43-4 and SA43-5, respectively. To ensure the structural stability of the stem–loop, a complementary CG base pair was added to the respective ends and junctions, with SA43-5 being connected into a whole by the CG base pairs. SA43-4 was further divided into two segments, SA43-4-1 and SA43-4-2, to assess the contributions of the flanking regions to the overall binding affinity. At the same time, the cavity structure of the stem–loop was removed, and the 16 nt flanking and junction parts were retained and designated as SA43-6. Among them, SA43-5 showed the strongest binding affinity among all variants. Therefore, SA43-5 was identified as the core binding sequence for *S. aureus* (Figure 1E).

### 3.2. Aptamer in Milk Matrix-Adapted SELEX

The tailored *S. aureus* aptamer has a length of 14 nt, and studies have shown that the deletion of nucleotides in aptamers can improve their affinity [33]. However, excessively short sequences may lead to reduced sequence specificity. Differences between screening conditions and actual application environments can also affect the binding of aptamers to targets. To enhance the detection performance of aptamers in milk samples, milk can be introduced during the screening process to simulate real application scenarios [34]. The tailored core sequence SA43-5 was incorporated into two structurally distinct random libraries for SELEX. Since the original SA43-5 sequence consisted of two 7nt segments, two libraries were designed accordingly: Library 1 featured a core sequence with 7 nt on each side flanking a 20 nt random region, while Library 2 contained a 14 nt core sequence flanked by 10 nt variable regions on both ends. Unique primer-binding sequences were introduced at the 5′ and 3′ ends of both libraries to prevent cross-contamination during amplification.

Negative SELEX using non-target bacterial strains was introduced in rounds 4, 7, and 9 to enhance the specificity of the screening. Rounds 8 to 10 were conducted under milk matrix conditions to improve the aptamer’s adaptability and selective recognition in dairy product detection environments. To further enhance aptamer affinity, the optimized SA43-5 sequence underwent progressive screening over eleven rounds, during which the selection conditions were gradually tightened, including reduced bacterial concentrations, shortened incubation times, and increased washing frequencies to enrich for high-affinity candidates (Table S1).

After SELEX, the PCR products were sequenced and analyzed. From the sequencing results of the two libraries, the ten most highly enriched sequences were chosen for homology analysis and phylogenetic tree construction using the DNAMAN 9.0 software (Figure S1A,B). Due to the limited length of the random regions in the libraries, the selected sequences exhibited relatively high homology overall (Figure S1C,D). Three aptamers with significant differences were selected from each of the two libraries and designated as SA1-1, SA1-2, SA1-3, SA2-1, SA2-2, and SA2-3. These six aptamers were evaluated for their binding capabilities using qPCR, and SA2-1 demonstrated excellent target-binding capacity (Figure S2, Table 2).

### 3.3. Aptamer Performance Evaluation

**Figure 2:**
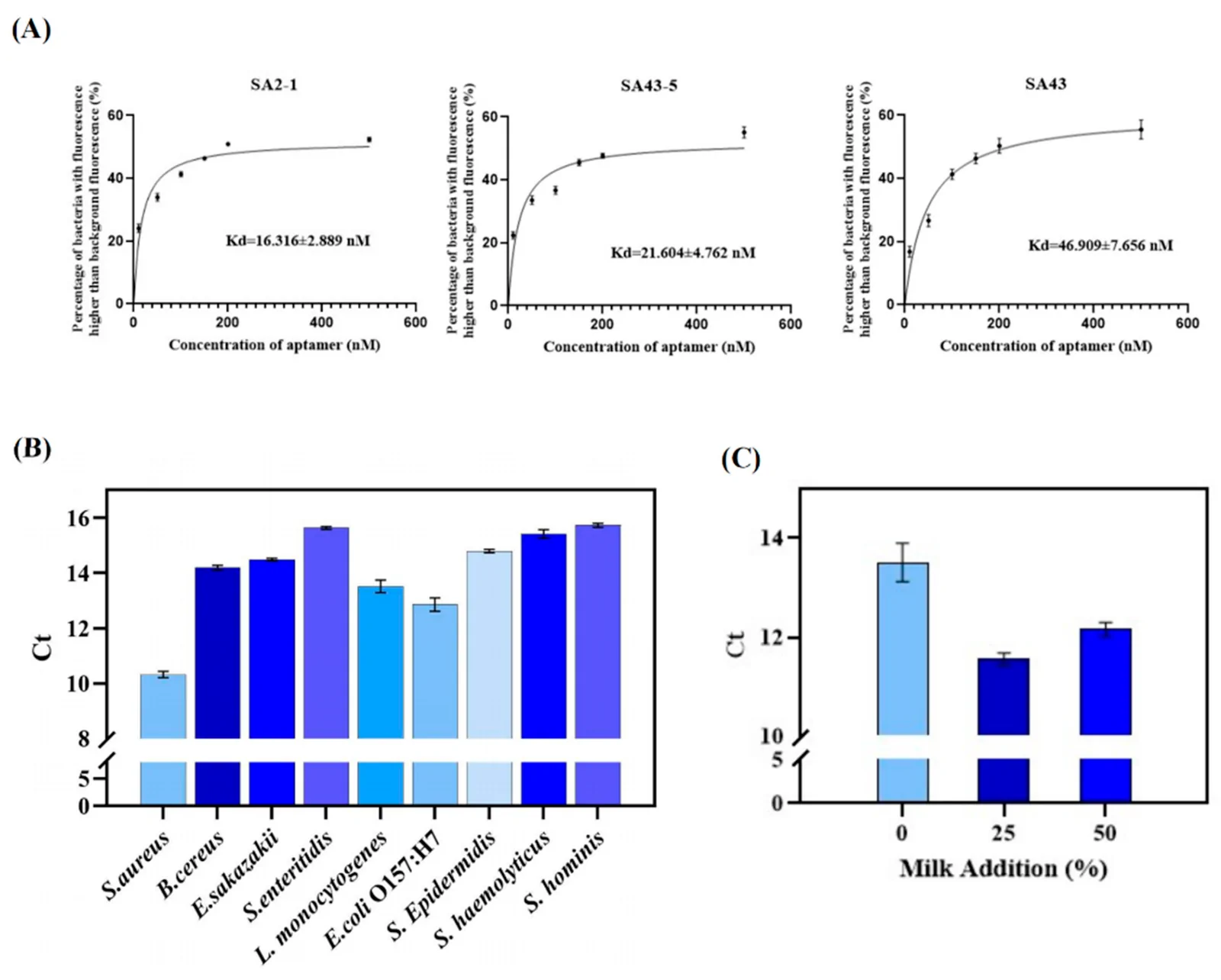
Aptamer performance validation. (**A**) Affinity validation of SA21, SA43-5, and SA43. (**B**) Specificity validation of SA2-1. (**C**) Binding validation of SA2-1 to *S. aureus* in deionized water, 25% milk, and 50% milk. Error bars indicate standard deviations from three independent biological replicates.

The affinities of SA2-1, the tailored core sequence SA43-5, and the original pre-tailored sequence SA43 were evaluated using flow cytometry. SA2-1 exhibited the best affinity, indicating that the affinity of the aptamer was improved after tailoring and SELEX. Therefore, the aptamer SA2-1 was incubated with *S. aureus* and five common foodborne pathogens from different genera (*E. sakazakii*, *L. monocytogenes*, *S. enteritidis*, *B. cereus*, and *E. coli* O157:H7), as well as three Staphylococcus species (*S. haemolyticus*, *S. epidermidis*, and *S. hominis*), followed by validation using qPCR. As shown in Figure 2A, *S. aureus* exhibited a lower Ct value, demonstrating that SA2-1 has specificity for *S. aureus*.

The affinity of SA2-1 for *S. aureus* in milk was evaluated. As illustrated in Figure 2B, samples with added milk all displayed lower Ct values. This result demonstrates that the tailored and optimized aptamer achieved the better recognition of *S. aureus* in milk matrices, confirming the effectiveness of matrix-adapted SELEX in optimizing aptamer performance for practical foodborne pathogen detection.

### 3.4. Construction of the Paper-Based Dual-Recognition Biosensor

**Figure 3:**
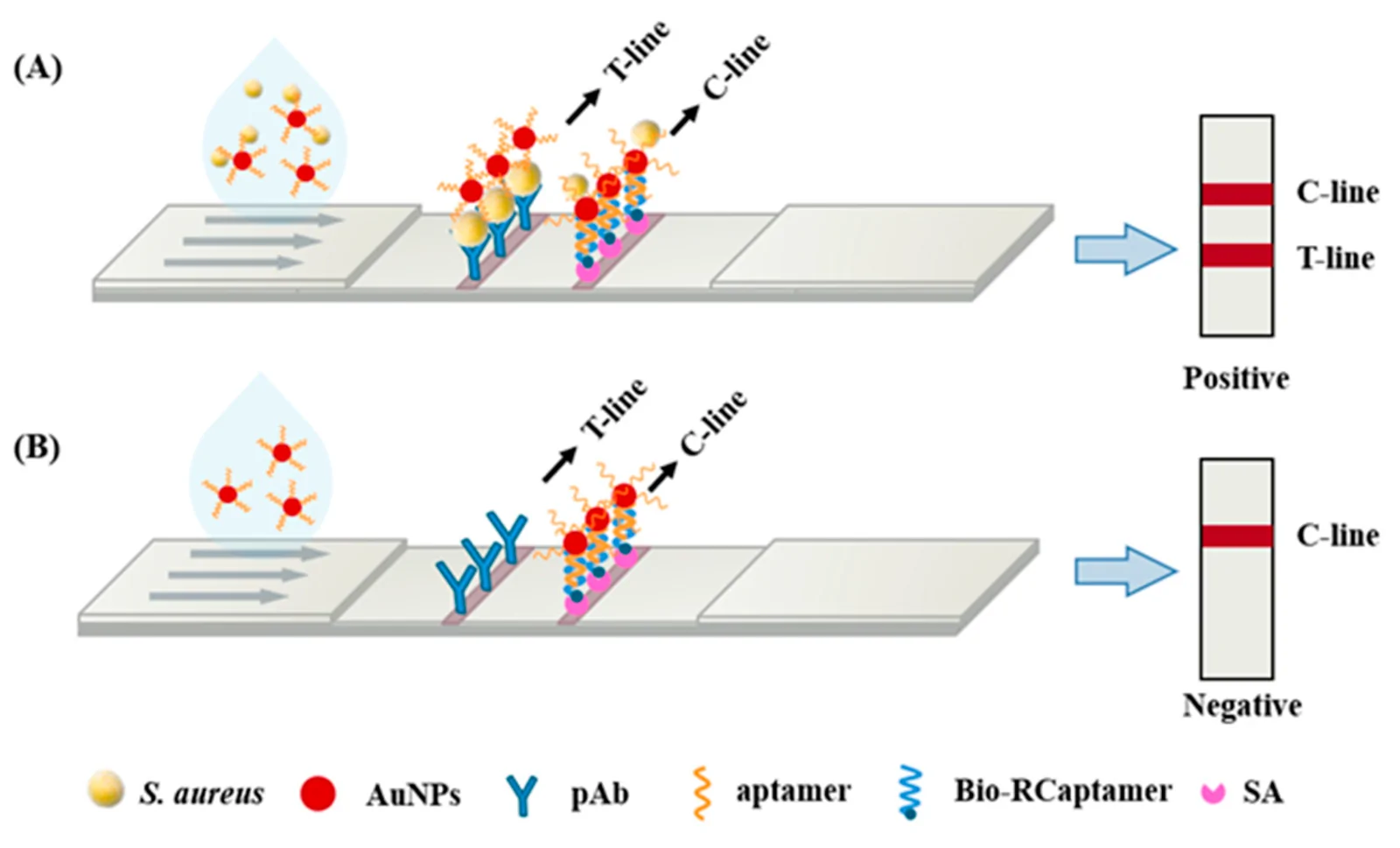
Schematic diagram of paper-based biosensor based on aptamer and antibody. (**A**) Positive results, (**B**) negative results.

In this study, a sandwich-type paper-based lateral flow biosensor with dual recognition was developed using aptamer SA2-1 selected through previous screening and polyclonal antibodies against Staphylococcus aureus. Figure 3 shows the overall schematic diagram of the designed biosensor. The first step is the conjugation of aptamer SA2-1 with AuNPs, which generates a colorimetric signal after the assay. The aptamer can be attached to the surfaces of AuNPs through covalent bonds between thiol and gold. The added BSA can block the unbound sites on the surfaces of AuNPs to prevent false-positive results. BSA also passivates the NC membrane to reduce the non-specific adsorption of gold conjugates during lateral flow. Unbound aptamers and BSA are removed by centrifugation, and the precipitate is resuspended to form detection probes. During detection, when the target *S. aureus* is present in the sample to be tested, the aptamer–AuNP probe will recognize and bind to the target, migrate to the strip along with the running buffer, and be captured by the immobilized polyclonal antibodies on the test line, resulting in the formation of a visible red signal on the T-line. As the target concentration decreases, the number of targets that can be simultaneously captured by the aptamer–AuNP probes and antibodies decreases, thereby weakening the color of the T-line. When there is no target in the sample to be tested, the aptamer–AuNPs will not be captured by the T-line when flowing through it. To ensure the validity of the test strip, the reverse complementary sequence of aptamer SA2-1 is immobilized on the C-line. Regardless of whether the test sample contains the target, the aptamer–AuNPs will be captured by the C-line.

### 3.5. Characterization and Optimization of Aptamer–AuNP Probes

**Figure 4:**
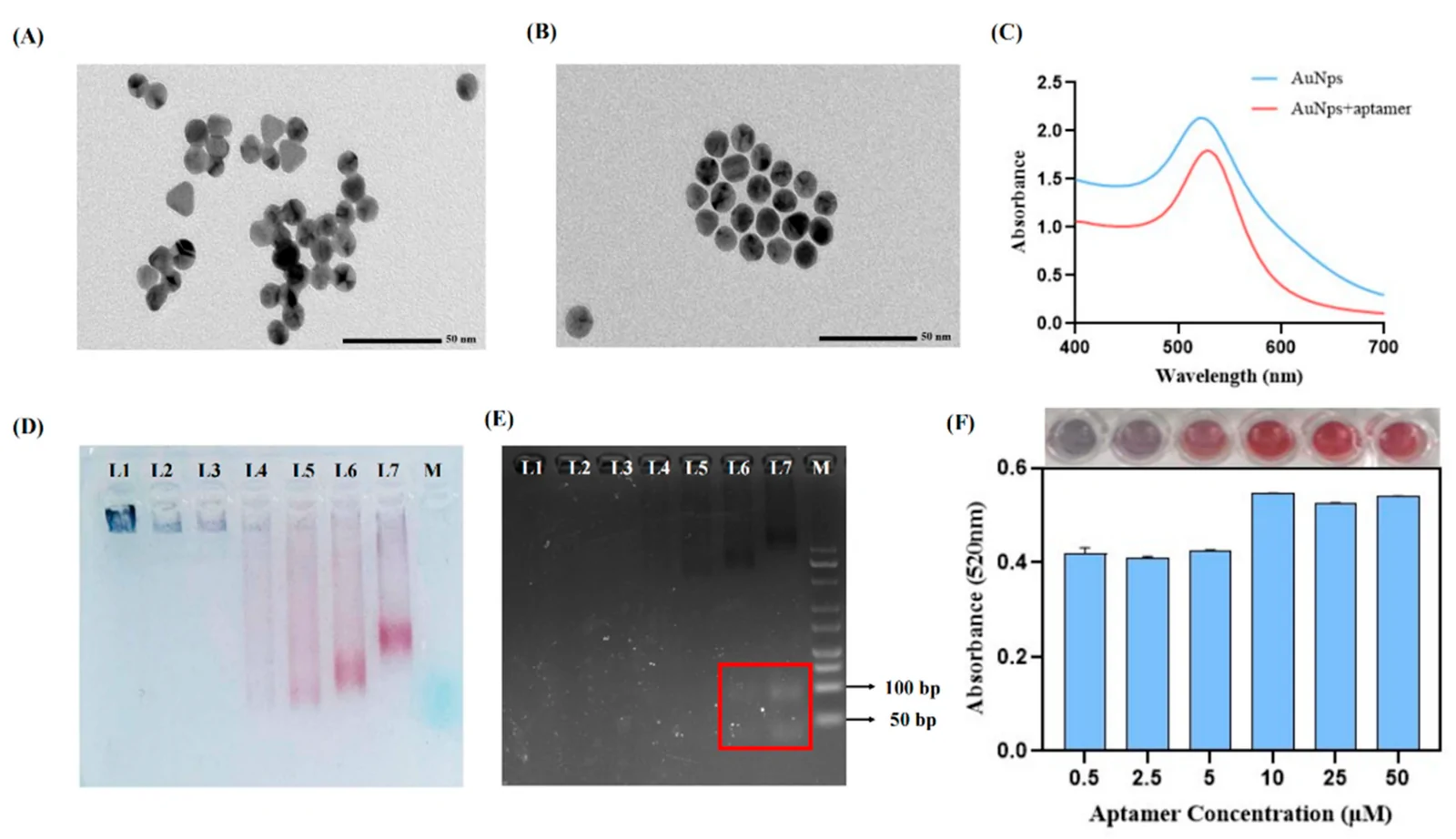
Characterization and optimization of aptamer–AuNP probes. (**A**) TEM image of AuNPs. (**B**) TEM image of aptamer–AuNP probes. (**C**) UV-Vis spectra of AuNPs and aptamer–AuNPs. (**D**) Agarose gel electrophoresis of AuNPs conjugated with aptamers at different concentrations. Lane 1: AuNPs without aptamer conjugation; Lanes 2–7: Aptamer concentrations of 0.5, 2.5, 5, 10, 25, and 50 μM, respectively; M: 50 bp DNA ladder. (**E**) Image of the gel from (**D**) under ultraviolet light. (**F**) Absorbance at 520 nm of aptamer–AuNP probes conjugated with different aptamer concentrations after the addition of 1 M NaCl. Error bars indicate standard deviations from three technical replicates.

As a key probe for target recognition, the aptamer must effectively bind to AuNPs to achieve efficient detection. TEM was used to describe the size distribution and morphology of the produced AuNPs. The TEM image in Figure 4A shows that the size of the AuNPs is concentrated at 12 ± 0.5 nm. As shown in Figure 4B, when the surfaces of the AuNPs are fully conjugated with the aptamer, the AuNPs change from a disordered aggregated state to an orderly arrangement, and there is a stable interval between each AuNP. When conjugated with aptamers, the maximum absorption wavelength of AuNPs exhibits a red shift, as shown in Figure 4C. Agarose gel electrophoresis can also verify the successful preparation of aptamer–AuNP probes. In Figure 4D, Lane 1 depicts AuNPs without aptamer conjugation, showing a dark blue band aggregated at the well, which is because the high ionic concentration of the electrophoresis solution causes the AuNPs to aggregate, making them unable to flow in the agarose gel. Lanes 4–7 show a wine-red band, proving that AuNPs can flow in the agarose gel after binding to aptamers.

The aptamer, as an important recognition molecule in this dual-recognition biosensor, was optimized for its concentration when conjugated to AuNPs to improve the detection sensitivity while controlling the detection cost. NaCl can disrupt the ionic environment, and, when aptamers are insufficient, it will cause the irreversible aggregation of the AuNPs. Therefore, different concentrations of aptamers were added to the AuNPs for conjugation, and 1 M NaCl was added to verify the conjugation effect. As shown in Figure 4F, the absorbance gradually increased with the increase in the aptamer concentration added to the AuNPs. From the agarose gel electrophoresis results in Figure 4E, it can be seen that, when the aptamer concentration reached 5 μM, the AuNPs no longer aggregated. With the added aptamer concentration increasing above 25 μM, a relatively bright band appeared at the bottom of the agarose gel, indicating that there was a large amount of free ssDNA in the aptamer–AuNP probe at this concentration, which suggested that the added aptamer concentration was excessive. Thus, 10 μM was selected as the optimal concentration for addition to AuNPs.

**Figure 5:**
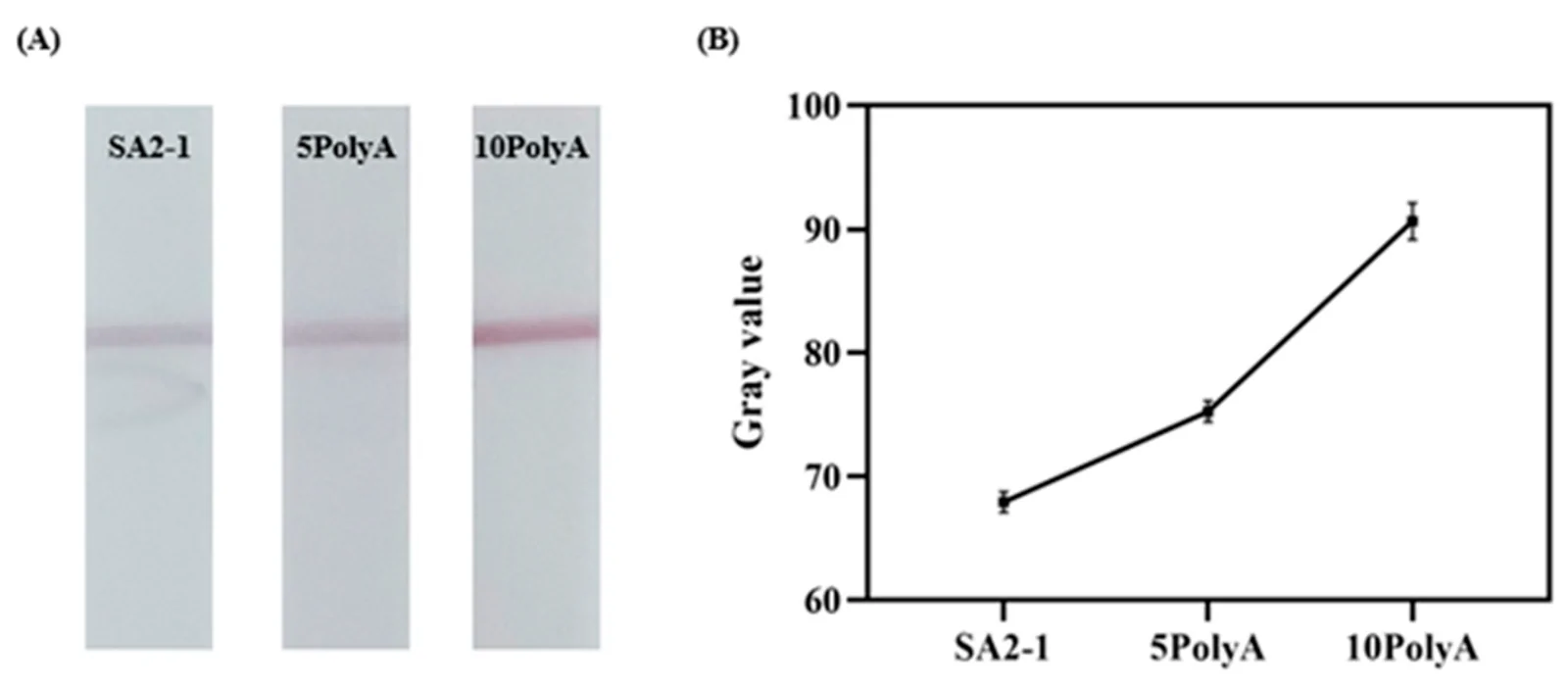
Effects of adding linkers to aptamers on the C-line. (**A**) Detection results of the original aptamer, aptamer with 5 polyA residues, and aptamer with 10 polyA residues; (**B**) gray values of linkers with different lengths on the C-line. Error bars indicate standard deviations from three technical replicates.

During the experiment, it was found that the color of the C-line on the test strip became significantly weaker after BSA blocking. To address this phenomenon, it was initially speculated that there might be two reasons: first, the BSA blocking concentration was inappropriate; second, during the blocking process, BSA molecules occupied the binding sites on the surface of the NC membrane and, at the same time, produced a steric hindrance effect, which hindered the specific binding between the aptamer complementary strands and the streptavidin–biotin complex immobilized on the C-line. To solve this problem, it was considered to introduce linkers of different lengths (5 polyA, 10 polyA, and no linker added) to the aptamers for optimization, and the C-line was the complementary sequence containing polyT of the corresponding length. The spatial distance of the aptamers was extended to reduce the impact of steric hindrance on the binding effect. The aptamers modified with linkers of different lengths were conjugated with AuNPs at the optimal concentration of 10 μM to prepare probes, which were then assembled into test strips for detection. The optimization effect was evaluated by observing the color change of the C-line. As shown in Figure 5A,B, the color of the C-line in the control group without a linker added was still relatively light; the color of the C-line in the 5 polyA linker group was somewhat darker but still not clear enough; and the color of the C-line in the 10 polyA linker group was significantly enhanced, and the band was uniform and bright. This indicates that the 10 polyA linker can effectively counteract the steric hindrance effect caused by BSA blocking, significantly improving the binding efficiency between the aptamer complementary strands and the immobilized substances on the C-line. Therefore, the aptamer modified with the 10 polyA linker was selected for subsequent experiments.

### 3.6. Optimization of Paper-Based Biosensor Preparation Conditions

**Figure 6:**
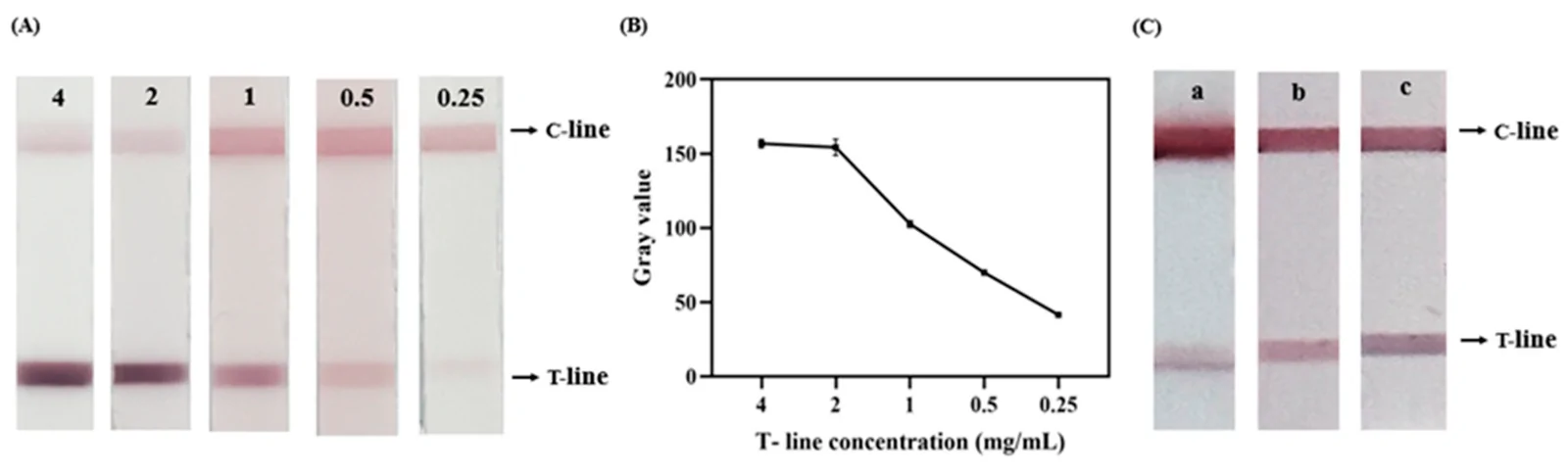
Condition optimization of the paper-based biosensor. (**A**) Detection results for T-line coated antibody concentration optimization; (**B**) analysis of grayscale values of T-line at different concentrations; (**C**) detection results with different running buffers: a: 10 mM Tris-HCl buffer containing 1% Tween 20, 0.5% Triton-X100, 0.25% PEG2000, and 2% sucrose, pH 7.4; b: 10 mM PBS buffer containing 1% Tween 20, 0.5% Triton-X100, 0.25% PEG2000, and 2% sucrose, pH 7.4; c: 10 mM PBS buffer containing 1% Tween 20, 0.5% Triton-X100, and 2% sucrose, pH 7.4.

As another important molecule for recognizing targets, optimizing the concentration of antibodies on the T-line is crucial [35]. An excessively high antibody concentration can cause the abnormal aggregation of AuNPs and irregular zigzag coloring at the T-line, while an overly low concentration results in weakened colorimetric signals. As can be seen from Figure 6A,B, when the antibody concentration was 4 mg/mL and 2 mg/mL, the excessively high concentration caused the aptamer–AuNP probes to aggregate at the T-line, making them unable to flow to the C-line. The antibody at a concentration of 1 mg/mL produced the optimal visual effect, with a bright and uniform color.

Optimizing the buffer system is essential to ensure a proper flow rate, minimize background interference, and facilitate the specific binding of targets to recognition molecules [36]. PBS buffer and Tris-HCl buffer were used as the main buffer systems to maintain a stable ionic environment. The surfactants Tween-20 and Triton X-100 were added to promote the smooth flow of the fluid. Since the test sample produced non-specific background signals after flowing through the test strip, PEG2000 was added to reduce such interference. As shown in Figure 6C, comparative tests on buffer systems indicated that the Tris-HCl buffer darkened the color of the gold nanoparticles and caused aggregation, which disrupted the conjugation system of the aptamer–AuNP probes. In contrast, the PBS buffer system effectively maintained the dispersion stability of the probes and significantly suppressed non-specific adsorption, thereby presenting the cleanest background and the clearest T-line and C-line.

### 3.7. Analytical Performance of the Paper-Based Dual-Recognition Biosensor

**Figure 7:**
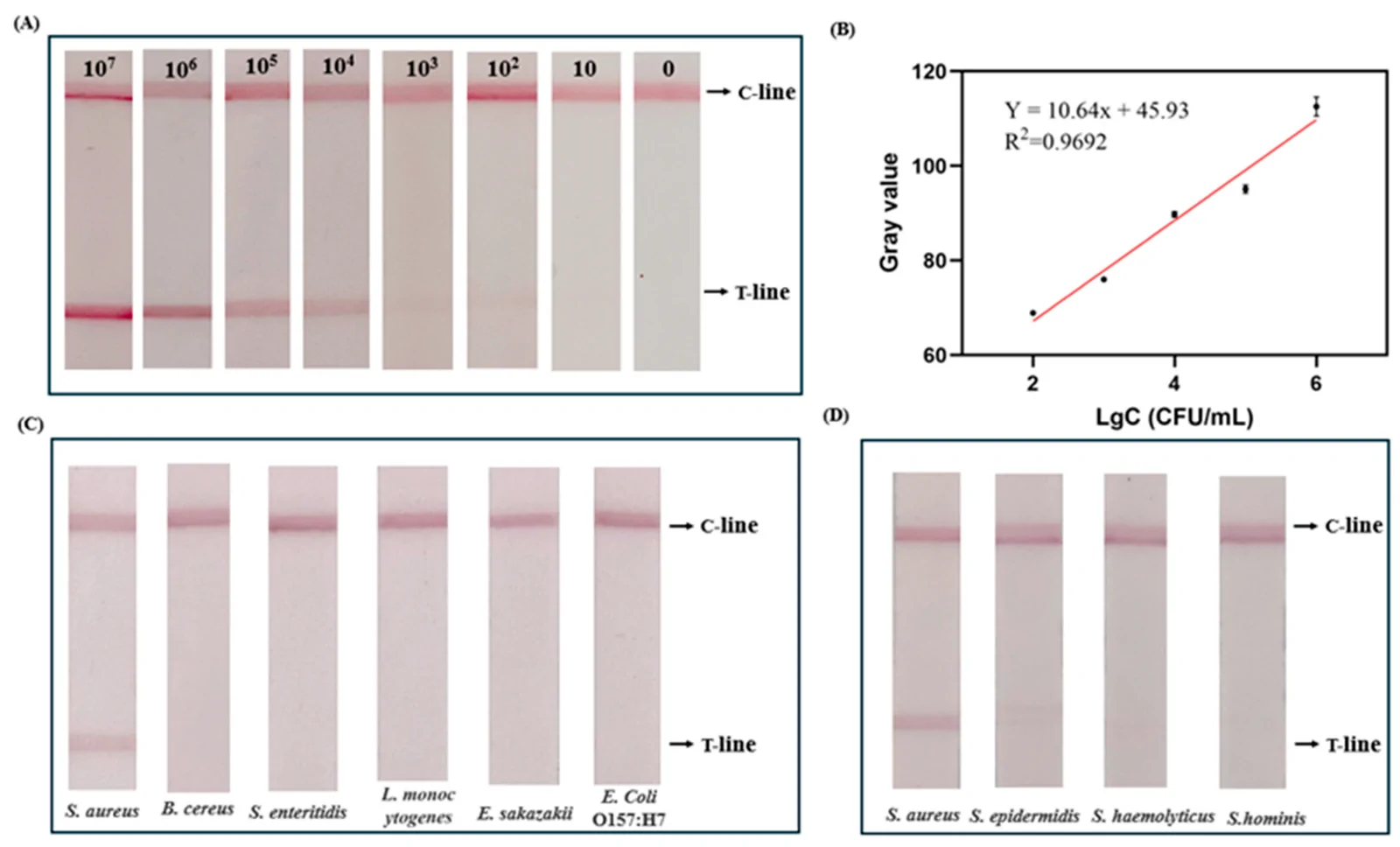
Analytical performance of the paper-based dual-recognition biosensor. (**A**) Sensitivity evaluation across a bacterial concentration range of 10^7^ to 0 CFU/mL, showing visible red bands at the test line down to 10^2^ CFU/mL. (**B**) Linear regression analysis of grayscale values versus the logarithmic bacterial concentration (lgC) in the range of 10^2^–10^6^ CFU/mL. Y = 10.64x + 45.93, R^2^ = 0.9692. (**C**) Specificity evaluation of *S. aureus* versus six other common foodborne pathogens: *B. cereus*, *E. sakazakii*, *S. enteritidis*, *L. monocytogenes*, and *E. coli* O157:H7. (**D**) Specificity evaluation of *S. aureus* versus three *Staphylococcus* strains: *S. epidermidis*, *S. haemolyticus*, and *S. hominis*.

Under optimized conditions, the constructed paper-based biosensor was used to detect *S. aureus* in solution. A suspension of *S. aureus* with an initial concentration of 10^7^ CFU/mL was serially diluted tenfold, mixed with running buffer, and then added to the sample pad of the test strip for detection. As the bacterial concentration decreased, the color intensity at the T-line gradually weakened. As shown in Figure 7A, when the concentration reached 10^2^ CFU/mL, a visible red band could still be observed on the T-line. However, when the concentration was further reduced to 10 CFU/mL, the red band was no longer visible.

Specificity evaluation was performed using *S. aureus*, six non-homologous foodborne pathogens, and three Staphylococcus strains at a concentration of 10^5^ CFU/mL. As shown in Figure 7C,D, only *S. aureus* produced a distinct red band at the T-line, with no observable cross-reactivity with other strains, confirming the high specificity for the target.

Furthermore, we compared our method with existing methods in the literature. As shown in Table 2, this aptamer and antibody-based dual-recognition biosensor demonstrates superior detection advantages. While electrochemical immunosensors and fluorescent aptasensors can achieve lower detection limits, they require prolonged incubation times, complex sample preprocessing, and sophisticated laboratory instrumentation. Similarly, lateral flow strips integrating isothermal amplification or CRISPR systems also rely on specific temperature control devices. In contrast, this method operates entirely without sample pre-processing or sophisticated instrumentation, matching or surpassing the sensitivity of conventional single-recognition colorimetric strips while delivering rapid results directly in milk matrices within 15 min.

**Table 2:** Comparison of the method proposed in this study with other reported methods for *S. aureus*.

Detection Method	Time (min)	LOD (CFU/mL)	Signal Output	Reference
Dual-recognition fluorescent immunochromatographic strip based on IgG and antibiotic	40	10^4^	Fluorometer	[37]
Alloy nanolabel-based immunochromatographic test strip	5	1.5 × 10^3^	Visual	[38]
Isothermal amplification and CRISPR/Cas12a system-based assay	40	6.7 × 10^2^	Visual	[39]
Fluorescent aptasensor employing DNA walking and hybridization chain reaction dual amplification	180	10	Fluorometer	[40]
SERS sensor employing functionalized metal nanoparticles and aptamers	50	1.09	Raman Spectrometer	[41]
Paper-based biosensor utilizing an antibody and aptamer sandwich configuration	≤20	10^2^	Visual	This Work

### 3.8. Detection of S. aureus in Actual Milk Samples

To evaluate the practical applicability of the paper-based dual-recognition biosensor in real samples, *S. aureus* at a concentration of 10^7^ CFU/mL was artificially inoculated into milk. As shown in Table 3, after 5 h of incubation, the bacterial concentration was measured as 3 × 10^5^ CFU/mL using a conventional plate counting method. The sample was then subjected to tenfold serial dilutions to prepare test samples at three different concentration levels, and it was tested using the developed biosensor. As shown in Table 3, the found concentrations were calculated by substituting the grayscale values of the test strip T-lines into the standard calibration curve (Figure 7B, Y = 10.64x + 45.93). The recovery rate (%) was calculated using the formula (found concentration/plate count concentration) × 100%. The results showed that the recovery rates of the sensor ranged from 101.6% to 105.6%, and the relative standard deviations (RSDs) were below 10%, indicating that the sensor possesses high accuracy and reproducibility in actual complex dairy matrices.

## 4. Conclusions

In this study, a paper-based biosensor was successfully developed using aptamers and antibodies as dual-recognition elements for the rapid and sensitive detection of *Staphylococcus aureus* in milk. The aptamers were selected and optimized through sequence truncation and the SELEX process, and they demonstrated strong binding affinity and high specificity. These aptamers were able to recognize the target strain reliably even in complex milk matrices These characteristics make it especially practical for food safety screening in resource-limited environments. Future research will aim to enhance the stability of the biosensor under various environmental conditions and to extend its application to the detection of other foodborne pathogens. This study highlights the promising potential of dual molecular recognition strategies for rapid pathogen screening and provides a valuable technological approach for improving food safety and public health.

## Figures and Tables

**Table 1:** Summary of oligonucleotides utilized in the tailoring phase.

Name	Sequence (5′–3′)	Length (nt)	Reference
SA31	TCCCACGATCTCATTAGTCTGTGGATAAGCGTGGGACGTCTATGA	45	[29]
SA43	TCGGCACGTTCTCAGTAGCGCTCGCTGGTCATCCCACAGCTACGTC	46	
SA14	ACACCGCAGCAGTGGGAACGTTTCAGCCATGCAAGCATCACGCCCGT	49	[30]
SA17	TCCCTACGGCGCTAACCCCCCCAGTCCGTCCTCCCAGCCTCACACCGCCACCGTGCTACAAC	64	[31]
RAB1	CGGGTGGGCTCCAATATGAATCGCTTGCCCTGACGCTATCT	43	[32]

**Table 3:** Actual sample testing.

Sample	Conventional Plate Counting Method (CFU/mL)	Found (CFU/mL)	Recovery (%)	RSD (%)
1	3 × 10^3^	3.16 × 10^3^	105.6	9.49
2	3 × 10^4^	3.05 × 10^4^	101.6	4.79
3	3 × 10^5^	3.14 × 10^5^	104.7	5.81

## Data Availability

The original contributions presented in this study are included in the article. Further inquiries can be directed to the corresponding author.

## Supplementary Materials

The following supporting information can be downloaded at: https://www.mdpi.com/article/10.3390/bios16080417/s1, Table S1: Relevant reaction conditions during SELEX; Table S2: Summary of oligonucleotides utilized in the tailoring phase; Table S3: Summary of oligonucleotides utilized in the SELEX; Figure S1: Sequence homology analysis and phylogenetic tree of aptamer candidates; Figure S2: Binding concentration analysis of SELEX candidate aptamers evaluated via qPCR; Figure S3: Time-course study of color development and signal stabilization on the paper-based biosensor.
