# Supplementary material for: Paper-Based Biosensor Using Dual-Recognition Molecules for Detection of Staphylococcus aureus in Milk
Source: Biosensors (Basel). 2026 Aug 3;16(8):417. doi: 10.3390/bios16080417 (PMC13511570; doi:10.3390/bios16080417)
Supplement: Supplementary file 1 [file biosensors-16-00417-s001.zip › biosensors-4428362-supplementary.pdf]

SUPPORTING INFORMATION

TABLE OF CONTENTS

Table S1.: Relevant reaction conditions during SELEX

Table S2.: Summary of oligonucleotides utilized in the tailoring phase

Table S3.: Summary of oligonucleotides utilized in the SELEX

Figure S1.: Sequence homology analysis and phylogenetic tree of aptamer candidates.

Figure S2.: Binding concentration analysis of SELEX candidate aptamers evaluated via qPCR.

Figure S3.: Time-course study of color development and signal stabilization on the paper-based biosensor.

Table S1. Relevant reaction conditions during SELEX

Rounds	Bacterial addition (μL)	Incubation time (Min)	Washing times (Min)	Milk powder matrix addition (μL)
1	1000	90	3	0
2	1000	90	4	0
3	800	60	4	0
4	1000	90	0	0
5	800	90	4	0
6	500	60	4	0
7	1000	90	0	0
8	800	90	5	500
9	1000	40	0	500
10	500	90	5	500
11	1000	30	5	0

Table S2. Summary of oligonucleotides utilized in the tailoring phase

Applications	Name	Sequence (5′-3′)	Length (nt)
Affinity comparison	forward primer (FP)	GCAATGGTACGGTACTTCC	19
	reverse primer (RP)	TTAGCAAAGTAGCGTGCACTTTTG	24
Tailoring	SA17-1	TCCCTACGGCGCTAACCCCCCCAGTCCGTCC	31
	SA17-2	TCCCAGCCTCACACCGCCACCGTGCTACAAC	32
	SA17-3	GTCCGTCCTCCCAGCCTCACACC	23
	SA43-1	TCGGCACGTTCTCAGTAGCGCTC	23
	SA43-2	GCTGGTCATCCCACAGCTACGTC	25
	SA43-3	GCGCTCGCTGGTCATCCCACAGC	25
	SA43-4	CGCTGGTCATCCCACAGCG	19
	SA43-4-1	GCTGGTCAT	9
	SA43-4-2	TCCCACAGC	9
	SA43-5	GCGCTCGCTACGTC	14
	SA43-6	GCTCGCTGTCAGCTACG	17

Table S3. Summary of oligonucleotides utilized in the SELEX

Applications	Name	Sequence (5′-3′)	Modification	Length (nt)
Matrix-adapted SELEX	Library 1	GCGCTCGNNNNNNNNNNNNNNNNNNNNCTACGTC		80
	FP-Apt 1	CGTACGGTCGACGCTAGC		16
	RP-Apt 1	GGATCCGAGCTCCACGTG	5’P	14
	Library 2	NNNNNNNNNNGCGCTCGCTACGTCNNNNNNNNNN		34
	FP-Apt 2	CAGGTCCATCGAGTGGTAGGA		31
	RP-Apt 2	GTACGTTCAGGAGCAGTGCGA	5’P	
Candidate selection	SA1-1	CGTACGGTCGACGCTAGCGCGCTCGAAAAGAAAACAAACCACACACTACGTC<br>CACGTGGAGCTCGGATCC		110
	SA1-2	CGTACGGTCGACGCTAGCCACTCATCTATCGCGCCTAACGAAACAGCCCACAA<br>TATCCCTCCACGTGGAGCTCGGATCC		110
	SA1-3	CGTACGGTCGACGCTAGCCCTCGCCACCCTCTTCGACAACAAAGAGGCCCCT<br>CTCCACGTGGAGCTCGGATCC		110
	SA2-1	CAGGTCCATCGAGTGGTAGGACCACAAAAAAGCGCTCGCTACGTCGAAAAAA<br>AACTCGCACTGCTCCTGAACGTAC		110
	SA2-2	CAGGTCCATCGAGTGGTAGGAAAAGGCTCACGCGCTCGCTACGTCACAAAAA<br>AAATCGCACTGCTCCTGAACGTAC		108
	SA2-3	CAGGTCCATCGAGTGGTAGGACAAAATAAGAGCGCTCGCTACGTCAAAAAAT<br>AAATCGCACTGCTCCTGAACGTAC		108

Notes: Primer regions are underlined. Notes: Primer regions are underlined

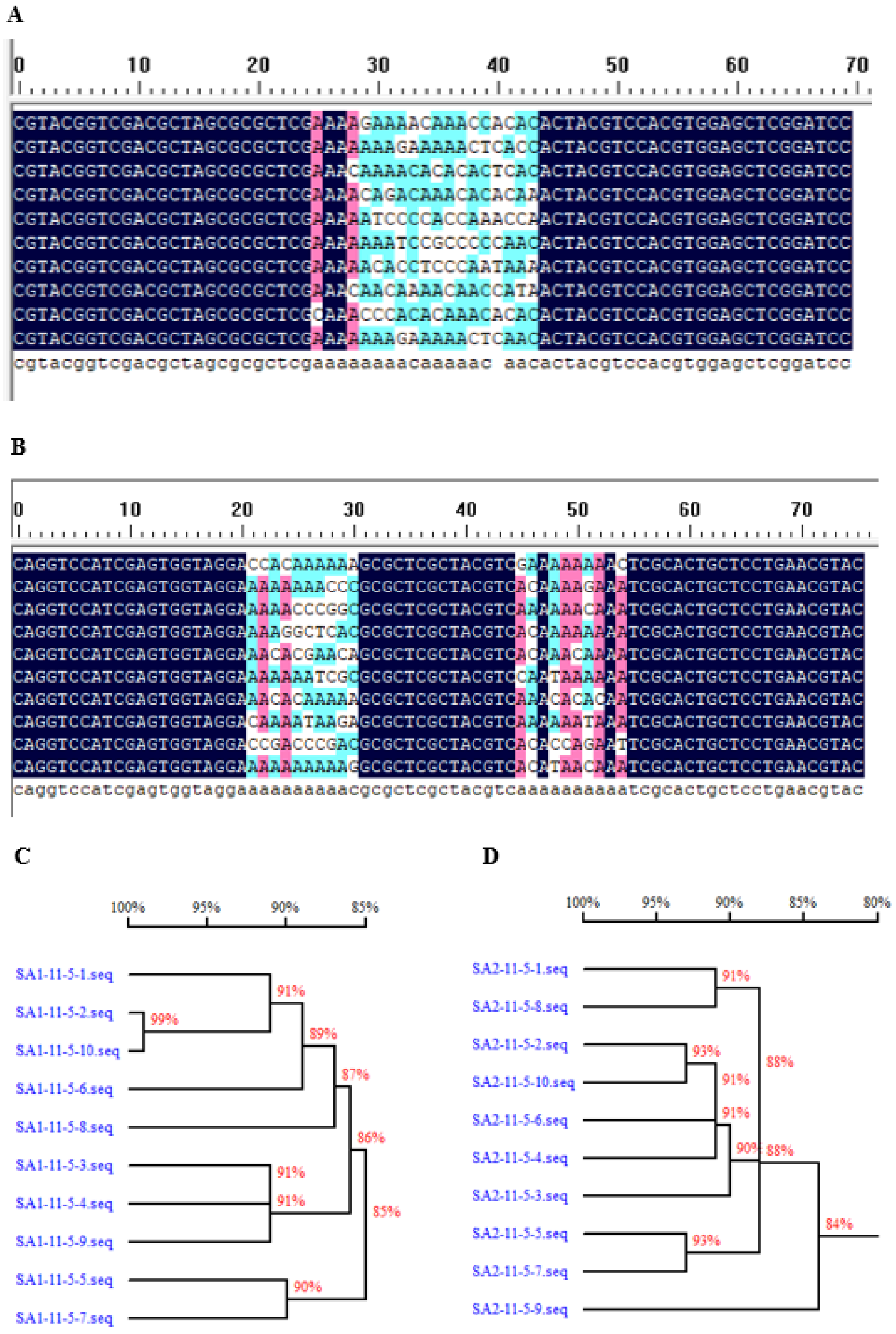

Figure S1. Sequence homology analysis and phylogenetic tree of aptamer candidates. (A) Sequence homology analysis of 10 aptamer candidates selected from Library 1 using DNAMAN software. Dark blue indicates complete sequence conservation at that position across all sequences; blue or red indicates partial conservation; no highlighting indicates no se−quence match at that position. (B) Sequence homology analysis of 10 aptamer candidates selected from Library 2. (C) Phylogenetic tree of the 10 aptamer candidates selected from Library 1. (D) Phylogenetic tree of the 10 aptamer candidates selected from Library 2.

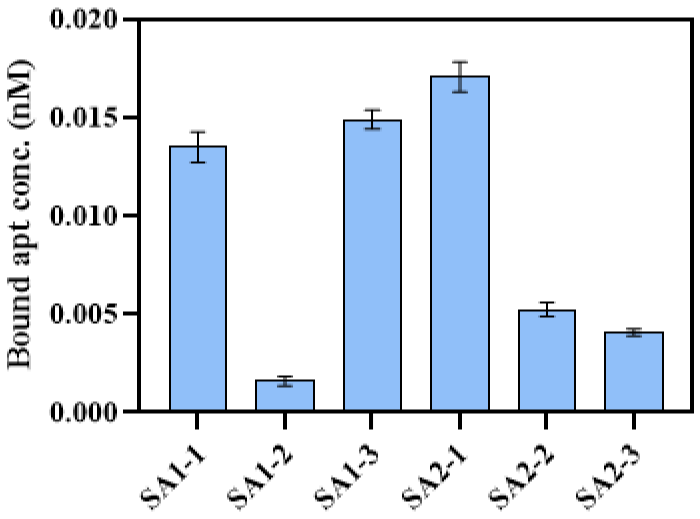

Figure S2. Binding concentration analysis of SELEX candidate aptamers evaluated via qPCR. Error bars indicate standard deviations from three independent technical replicates.

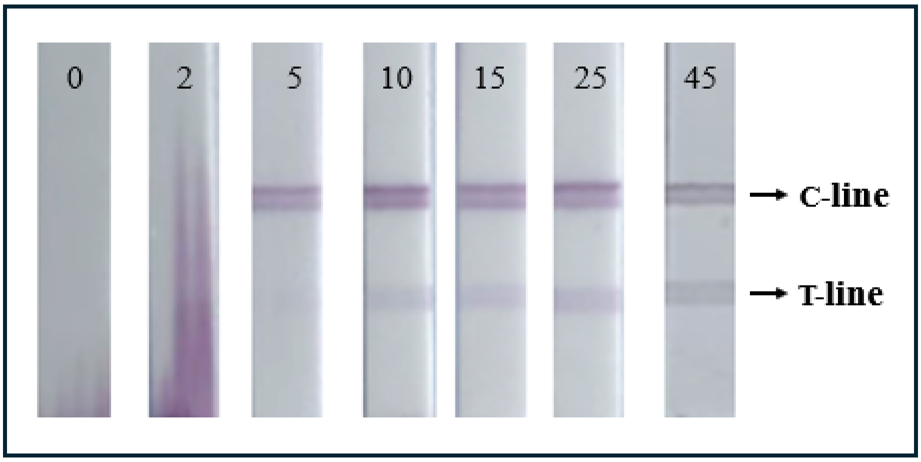

Figure S3. Time−course study of color development and signal stabilization on the paper−based biosensor.
